# Letter to the editor: Chikungunya infection in travellers to Thailand: additional United Kingdom cases identified by specialist laboratory

**DOI:** 10.2807/1560-7917.ES.2019.24.24.19-00333

**Published:** 2019-06-13

**Authors:** Kirsten MacGregor, Gemma Buckland-Merrett, Daniel Bailey, Emma Aarons

**Affiliations:** 1 Rare and Imported Pathogens Laboratory, Public Health England, Porton, Salisbury, United Kingdom; 2 National Infection Service, Public Health England, Colindale, London, United Kingdom

**Keywords:** United Kingdom, Thailand, vector-borne infections, chikungunya virus, outbreaks, laboratory surveillance, sentinel surveillance, syndromic surveillance, travel

**To the editor:** As the specialist laboratory providing chikungunya virus (CHIKV) diagnostics to the National Health Service (NHS) across the United Kingdom (UK), we read with interest the rapid communication by Javelle et al. [1] regarding the chikungunya outbreak in Thailand at the beginning of 2019.

Alongside the two UK cases reported in the paper, we diagnosed a further two CHIKV infections in travellers returning to the UK from Thailand in the same timeframe (the molecular assay used is based on the method of Edwards et al. [2], rather than Pastorino et al. [3], as was incorrectly indicated in the published paper). Case 1 recovered completely within 3 weeks, but case 2, an older individual, has been referred to rheumatology for management of persistent arthralgia, which has improved after a course of steroids (Table).

**Table:** Overview of two further confirmed chikungunya cases among travellers returning from Thailand to the UK, January–February 2019 (n = 2)

Case	Reporting Country	Place visited	Period of exposure	Age^a^ (years)	Underlying conditions	Date of onset	Clinical acute symptoms	Outcome	Recovery status as at 9 May 2019	Diagnosis method (positive tests)
1	UK	Unknown	December 2018–January 2019	40	None	January 2019	Rash, fever, headache, arthralgia, myalgia	Recovered	Yes	CHIKV RT-PCR [2] and anti-CHIKV IgM ELISA (Euroimmun, Lubeck, Germany) on day 5
2	UK	Koh Lanta	Unknown– February 2019	60	None	Unknown	Fever, headache and synovitis of small and large joints	Persistent arthralgia	No	CHIKV RT-PCR [2]

UK: United Kingdom.

^a ^Rounded to the nearest 5-year group.

The two additional cases we diagnosed were not reported to any of the data collection networks for the surveillance of travel-related illness. This indicates a limitation of surveillance networks that receive their data from the medical centres where patients are clinically assessed, rather than the laboratories that diagnose the infections. With this in mind, it is likely that there were more chikungunya infections from Thailand diagnosed in specialist laboratories across Europe in the first two months of 2019 than were reported to EuroTravNet/GeoSentinel or TropNet.

Currently, national public health bodies across the European Union/ European Economic Area (EU/EEA) report laboratory diagnoses of CHIKV infection to the European Centre for Disease Prevention and Control on an annual basis via the European Surveillance System (TESSy) [4]. Timely information on the numbers and sources of imported infections is therefore not available. We wonder whether a laboratory-based system of monthly or semi-monthly notifications of imported infections such as dengue and chikungunya could be considered.

TESSy is a system for the collection, analysis and dissemination of data on communicable diseases. Indeed, there is precedent for TESSy to be used for more regular reporting of data; at the time of the Zika virus (ZIKV) outbreak in 2016, ZIKV diagnoses data from across the EU/EEA were collected weekly for real-time surveillance. Direct laboratory notifications might contribute less clinical data than clinician notifications of laboratory-diagnosed imported infections such as chikungunya and dengue; nevertheless, we suggest that the routine availability of timely information regarding the countries of origin of CHIKV and dengue virus infections imported into Europe would be invaluable to providers of both pre-travel advice and healthcare for returning travellers.
